# The modeled structure of the RNA dependent RNA polymerase of GBV-C Virus suggests a role for motif E in *Flaviviridae *RNA polymerases

**DOI:** 10.1186/1471-2105-6-255

**Published:** 2005-10-14

**Authors:** François Ferron, Cécile Bussetta, Hélène Dutartre, Bruno Canard

**Affiliations:** 1 Architecture et Fonction des Macromolécules Biologiques, UMR 6098 CNRS et Université Aix-Marseille I et II, ESIL, Campus de Luminy, 13288 Marseille Cedex 09, France; 2 Boston Biomedical Research Institute, 64, Grove St, Watertown 02472, MA, USA

## Abstract

**Background:**

The *Flaviviridae *virus family includes major human and animal pathogens. The RNA dependent RNA polymerase (RdRp) plays a central role in the replication process, and thus is a validated target for antiviral drugs. Despite the increasing structural and enzymatic characterization of viral RdRps, detailed molecular replication mechanisms remain unclear. The hepatitis C virus (HCV) is a major human pathogen difficult to study in cultured cells. The bovine viral diarrhea virus (BVDV) is often used as a surrogate model to screen antiviral drugs against HCV. The structure of BVDV RdRp has been recently published. It presents several differences relative to HCV RdRp. These differences raise questions about the relevance of BVDV as a surrogate model, and cast novel interest on the "GB" virus C (GBV-C). Indeed, GBV-C is genetically closer to HCV than BVDV, and can lead to productive infection of cultured cells. There is no structural data for the GBV-C RdRp yet.

**Results:**

We show in this study that the GBV-C RdRp is closest to the HCV RdRp. We report a 3D model of the GBV-C RdRp, developed using sequence-to-structure threading and comparative modeling based on the atomic coordinates of the HCV RdRp structure. Analysis of the predicted structural features in the phylogenetic context of the RNA polymerase family allows rationalizing most of the experimental data available. Both available structures and our model are explored to examine the catalytic cleft, allosteric and substrate binding sites.

**Conclusion:**

Computational methods were used to infer evolutionary relationships and to predict the structure of a viral RNA polymerase. Docking a GTP molecule into the structure allows defining a GTP binding pocket in the GBV-C RdRp, such as that of BVDV. The resulting model suggests a new proposition for the mechanism of RNA synthesis, and may prove useful to design new experiments to implement our knowledge on the initiation mechanism of RNA polymerases.

## Background

The *Flaviviridae *virus family comprises three genera pestivirus, hepacivirus, and the large group of flavivirus. HCV causes acute and chronic hepatitis that may lead to cirrhosis and/or liver cancer. HCV is a major human pathogen, with 170 million people infected worldwide and 3 to 4 million of newly infected people each year [1]. Despite its large socio-economic impact, there is neither a vaccine nor an efficient, side-effect free therapy against this virus. Thus, the identification of potent drugs would be a major public health achievement. However, convenient small-animal models or productively infected cell systems to study HCV are still lacking. Consequently, compounds are often directly validated in HCV infected chimpanzees, or in cultured cells infected with related, surrogate viruses such as pestiviruses. The latter are animal pathogens showing similarity to hepaciviruses and flaviviruses [2] in genome structure, replication strategy, and individual gene products.

The RNA-dependent RNA polymerase (RdRp) is an enzyme playing a key role in the RNA replication process. Despite the increasing number of studies on the characterization of RdRp activity and structure, the precise molecular mechanism remains unclear. The postulated RNA replication process is a two-step mechanism. First, the initiation step of RNA synthesis begins at or near the 3' end of the (+) RNA template by means of a primer-independent (*de novo*) mechanism [3]. The *de novo *initiation consists in the addition of a nucleotide tri-phosphate (NTP) to the 3'-OH of the first initiating NTP. During the following so-called elongation phase, this nucleotidyl transfer reaction is repeated with subsequent NTPs to generate the complementary RNA product [3-6].

**Figure 1:**
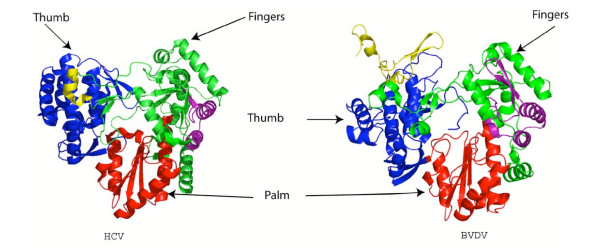
**Ribbon representation of the RdRp structure**. HCV and BVDV RdRps are represented with their different subunits and domains. The thumb is colored in dark blue and yellow, fingers are colored in green and purple and the palm is colored in red. The image was generated using PYMOL.

The structure of the RdRp of HCV (NS5B) has been determined [7,8]. It serves as reference in the uncovering of mechanism [9,10] and as link between structure and biochemical data for RNA polymerases [11]. The HCV polymerase shape resembles a semi-closed right hand and is made of three subdomains: fingers, palm and thumb (Figure 1). Computational and structural analysis of viral RdRp sequences has identified five universal motifs (F, A, B, C, E) located in (or close to) the palm (additional file 1). These motifs are both catalytic and structural. Fingers are made of a β-strand subdomain (four strands β1, β2, β4, β5 and an α-helix α1) and an α-helix rich subdomain (seven helices αA αB, αC, αD, αE, αF, αH). The palm is made of three stranded anti-parallel β-sheet (β3, β6, β7, and three helices (αG, αJ, αK). The thumb is mainly made of α-helices αN, αM, αL, αQ, αO, αP, αR) and a two-stranded antiparallel β-sheet (β10, β11) forming an extra structure called "the flap". The flap is proposed to play a role in the initiation mechanism, allowing only ssRNA to access the active site, and helping the correct positioning of the first two nucleotides [8].

Based on the structure of HCV RdRp solved in complex with NTPs [8], several GTP and NTP binding sites have been proposed. One is located behind the thumb, in a pocket on the surface of the structure, and has been called the allosteric (or surface) GTP binding site. The second one is in the catalytic cavity, where NTP can bind at various sites called P (priming), C (catalytic), and I (interrogating). Recently, the crystal structure of the RdRp of Bovine viral diarrhea virus (BVDV) has been published [12]. Another GTP binding site was found in the catalytic site, distinct from the P, C, and I sites of HCV NS5B. In the latter structure, this site corresponds to a cavity filled with water.

BVDV and HCV polymerases share a similar fold (Figure 1), but exhibit differences in the fingers and thumb subdomains due to differences in the number of secondary structure elements. As for the HCV polymerase, the shape of the BVDV polymerase is a semi-closed right hand made of fingers, palm, and thumb. Fingers are made of eleven β-strands, and twelve α-helices. The palm domain shows great conservation with the HCV palm domain. It consists of four strands forming a central β-sheet surrounded by three α-helices. The thumb contains height α-helices and five β-strands. The flap is lacking in BVDV RNA polymerase although Choi & et al [12] proposed that two β-strands with their connecting loops play the same role.

A number of structural differences in the flap and other subdomains raise the question of the relevance of BVDV as a surrogate model to discover HCV RNA polymerase inhibitors. Few years ago, "GB" viruses were identified and characterized as *Flaviviridae *agents leading to hepatitis [2] but not belonging to hepacivirus. Previous phylogenetic studies of GBV viruses were based on NS3 sequence comparisons [2]. Out of the three GB viruses identified so far, namely GBV-A, -B, and -C, two of them (GBV-A and GBV-B) are most likely monkey viruses while GBV-C can infect humans. HCV and GB virus genomes are organized in a similar way [13,14]. This similarity has been extended to the functional level with the characterization of the polymerase activity carried out by NS5B [15,16]. GBV-C virus allows a productive infection of cultured cells, that makes it a relevant alternate virus to be used as a model for HCV antiviral drug screening. In this study, we show using a NS5B-based phylogenetic analysis that GB viruses indeed carry the closest known RdRp to HCV in *Flaviviridae*. We have built a structural model for the GBV-C polymerase, which allows comparative analysis with HCV, and BVDV polymerase. Results presented in this paper suggest a novel model for the initiation of RNA synthesis in *Flaviviridae*. Due to its phylogenetic closeness to HCV, GBV-C might be an alternate and more relevant surrogate viral system than BVDV to HCV. Finally, the GBV-C polymerase model proposed in this study might help drug discovery and guide the characterization of the RNA polymerization mechanism.

## Results and Discussion

### Sequence analysis and phylogenic distribution

To compare *Flaviviridae *RdRps, we have used the set of sequences defined in VaZyMolO [17], that includes all sequences of completely sequenced viral genomes (Table 1). 

**Figure 2:**
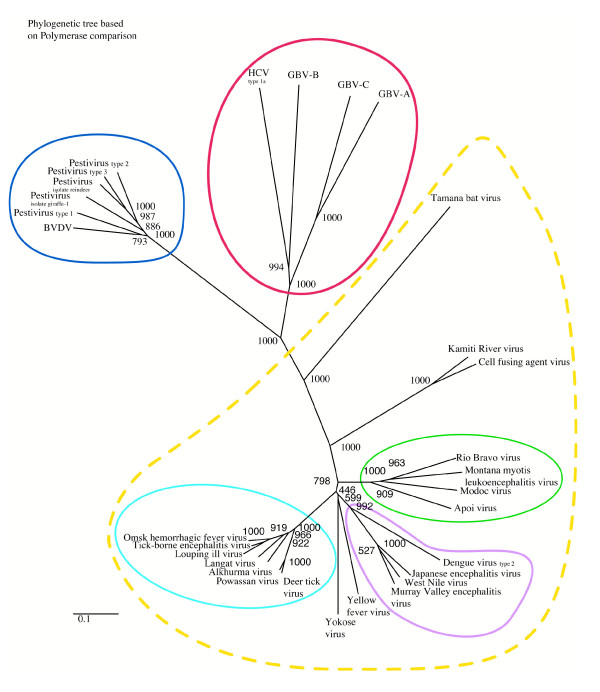
**The phylogenetic tree of *Flaviviridae *RdRps**. Numbers at nodes indicate the statistical support of the branching order by bootstrap criteria. The bar at the bottom of the phylogram indicates the evolutionary distance, to which the branch lengths are scaled based on the estimated divergence. The dashed yellow line indicates flavivirus genus, the blue line indicates pestivirus genus and the red line indicates hepacivirus genus.

The polymerase gene product alignment is based both on motif conservation and structural superimposition or conservation of secondary structures. We observe a great disparity depending on the genera of the compared sequences. Based on the alignment, a tree was derived (Figure 2 and additional file 1). Three major groups appear corresponding to the respective genus. Pestiviruses form a clear group distant from hepacivirus and flavivirus. This latter is the largest group of the family. It may be divided into several groups and isolated viruses reflecting adaptation. GB viruses cluster with hepacivirus in one group. This phylogenic distribution suggests that, in terms of a most relevant model polymerase useful in the screening of anti-viral drugs, GBV-C is closer to HCV than BVDV. The PSI-BLAST [18] search against non-redundant data bases (nrdb) using the GBV-C polymerase as an input sequence converges after one iteration and retrieves the HCV polymerase only, with an E-value of 9510^-59^.

**Table 1:** A listing of *Flaviviridae*. Viruses used in the study, together with their correspondent VaZyMolO and NCBI accession numbers.

Flaviviridae			
Data Base Accession	Virus	NCBI Acc Protein	Genus

VaZy 268	Dengue virus type 2	NP_056776.1	
VaZy 270	Omsk hemorrhagic fever virus [Bogoluvovska]	NP_878909.1	
VaZy 345	West Nile virus	NP_041724.2	
VaZy 387	Kamiti River virus – isolate SR-82	NP_891560.1	
VaZy 389	Yokose virus [Oita 36]	NP_872627.1	
VaZy 506	Hepatitis C virus type 1a – isolate H77	NP_671491.1	
VaZy 508	Murray Valley encephalitis virus	NP_051124.1	
VaZy 509	Japanese encephalitis virus	NP_059434.1	
VaZy 510	Pestivirus type 1 [NADL]	NP_040937.1	
VaZy 511	Cell fusing agent virus	NP_041725.1	
VaZy 512	Yellow fever virus [Flavivirus (mosquito-borne)]	NP_041726.1	
VaZy 513	Hepatitis GB virus B	NP_056931.1	
VaZy 514	Bovine viral diarrhea virus genotype 2 [C413]	NP_044731.1	
VaZy 515	Pestivirus type 3 [X818; Clover Lane]	NP_620062.1	
VaZy 516	Tick-borne encephalitis virus	NP_043135.1	
VaZy 518	Powassan virus [LB]	NP_620099.1	
VaZy 519	Hepatitis GB virus C	NP_043570.1	
VaZy 520	Pestivirus type 2 [Eystrup]	NP_075354.1	
VaZy 521	Langat virus [TP21]	NP_620108.1	
VaZy 522	Louping ill virus [369/T2]	NP_044677.1	
VaZy 523	Deer tick virus [ctb30] – isolate CT95	NP_476520.1	
VaZy 524	Tamana bat virus	NP_658908.1	
VaZy 525	Hepatitis GB virus A	NP_045010.1	
VaZy 526	Modoc virus [M544]	NP_619758.1	
VaZy 527	Montana myotis leukoencephalitis virus	NP_689391.1	
VaZy 528	Rio Bravo virus [RiMAR]	NP_620044.1	
VaZy 529	Alkhurma virus [1176]	NP_722551.1	
VaZy 530	Apoi virus [ApMAR]	NP_620045.1	
VaZy 531	Pestivirus – isolate reindeer-1 V60-Krefeld	NP_620051.1	
VaZy 532	Pestivirus – isolate giraffe-1 H138	NP_620053.1	
	GB group		
	Pestivirus		
	Hepacivirus		
	Flavivirus		
			
		sub group	
			

### Homology modeling of the GBV-C Virus RNA Polymerase

**Figure 3:**
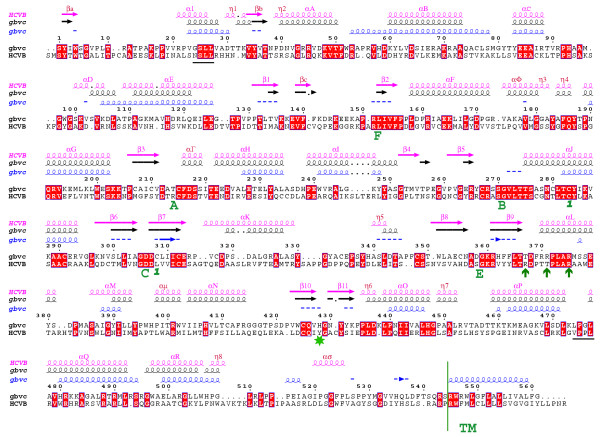
**Alignment of the structural template (HCV) and the sequence of GBV-C**. Sequence alignment of the HCV polymerase and the GBV-C polymerase. Identical amino acids are boxed in red. We superimposed secondary structure elements from the HCV polymerase in pink, the predicted structural elements of the GBV-C polymerase in blue and the secondary structure element of our final model in black. The HCV numbering according to [7] is given in pink. The numbering in dark red corresponds to the structure elements which have been observed with a better resolution. The dots in the alignment and structural elements (predicted or average) symbolise gaps. Green letters show universal motifs of RNA polymerases. Green arrows indicate amino acids involved in NTP binding. The green star indicates the amino acid supposed to stack the priming base. The numbering is that of the GBV-C polymerase. Numbers in dark green indicate cysteines involved in a putative disulfide bridge in our model. Residues forming the allosteric GTP binding site are underlined in black.

A sequence alignment of GBV-C and HCV polymerases is presented in Figure 3. It is based on sequence and structure comparison taking into account the prediction of secondary structure for GBV-C. In order to validate our method to predict the secondary structure, we have first used the HCV polymerase NS5B as a test sequence. Using the software PREDICT PROTEIN [19], 50% of the β-sheets and 84% of the α-helix are correctly predicted in the HCV polymerase, and using PSI-PRED [20] we obtain 87.5% of correctly predicted structural elements. Such prediction results make us confident with respect to the reliability of the GBV-C prediction. The secondary structure elements of HCV polymerase and the structural prediction of the GBV-C polymerase are superimposed on the sequence alignment shown in Figure 3. The comparison between the secondary structure elements observed in the HCV crystal structure and the prediction made for GBV-C polymerase (Figure 3) shows that β˜
 MathType@MTEF@5@5@+=feaafiart1ev1aaatCvAUfKttLearuWrP9MDH5MBPbIqV92AaeXatLxBI9gBaebbnrfifHhDYfgasaacH8akY=wiFfYdH8Gipec8Eeeu0xXdbba9frFj0=OqFfea0dXdd9vqai=hGuQ8kuc9pgc9s8qqaq=dirpe0xb9q8qiLsFr0=vr0=vr0dc8meaabaqaciaacaGaaeqabaqabeGadaaakeaacuaHYoGygaacaaaa@2E5C@ strands and α˜
 MathType@MTEF@5@5@+=feaafiart1ev1aaatCvAUfKttLearuWrP9MDH5MBPbIqV92AaeXatLxBI9gBaebbnrfifHhDYfgasaacH8akY=wiFfYdH8Gipec8Eeeu0xXdbba9frFj0=OqFfea0dXdd9vqai=hGuQ8kuc9pgc9s8qqaq=dirpe0xb9q8qiLsFr0=vr0=vr0dc8meaabaqaciaacaGaaeqabaqabeGadaaakeaacuaHXoqygaacaaaa@2E5A@ helices are almost perfectly superimposed, albeit small gaps are located in few α-helices or loops. The alignment shows 32% identity and 72% similarity. Insertions and deletions localize in loops primarily. The amino acid conservation in the fingers and palm is close to 40% identity. Both motifs (F, A, B, C, E) and the residues involved in the I site (Arg 45, Lys 48, Lys 145, and Arg 151) match very well. As in the crystal structure of the HCV polymerase where the 55 C-terminal amino acids are deleted, we did not include the last 47 amino acids at the GBV-C polymerase C-terminus.

**Figure 4:**
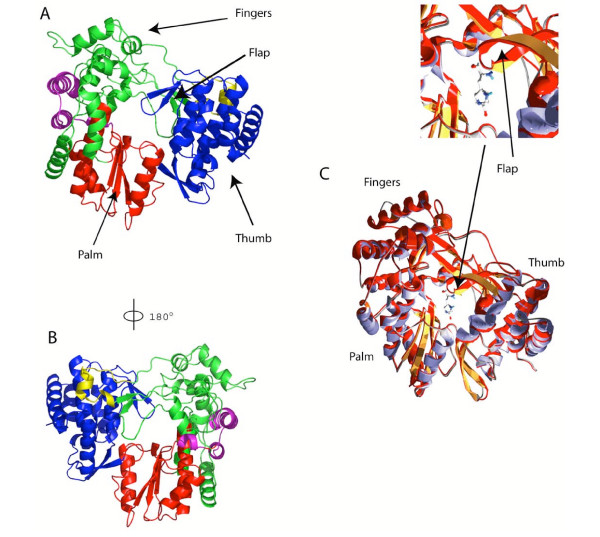
**Structural comparison of GBV-C and HCV RdRps**. A: The model of the GBV-C polymerase is presented as a front view highlighting the Flap and the histidine residue pointing to the catalytic site. The color scheme is the same as in Figure 1. Images were generated using POV-RAY. B: a 180° rotation view of the GBV-C model show in A. Images were generated using POV-RAY. C: Superimposition of the X-ray structure of the HCV polymerase (in red) and the GBV-C polymerase model (in purple and yellow). A zoomed view of the Flap region is presented in the upper side box in order to highlight the perfect superimposition of the aromatic ring of the histidine found in the GBV-C polymerase and the tyrosine found in the HCV polymerase. Images were generated using POV-RAY.

Both the sequence alignment and predicted secondary structure shown in Figure 3 were used in SWISS-MODEL (see methods) [21] to build and refine the GBV-C polymerase model (Figure 4). Alternative models were also generated using SCRWL [22], 3D-JIGSAW [23-25] and MODELLER [26] and evaluated using VERIFY3D [27]. The results are presented in the additional files 4 and 5. All models are evaluated as good by VERIFY3D [27], and are very similar, although some differences exist in flexible loops. Key residues of the active site are perfectly superimposed, unlike side chains because of their flexibility (additional file 4). These similar results make us very confident of the reliability of the GBV-C polymerase model, and for clarity, we will focus on the model generated by SWISS-MODEL [21]. The modeled structure was then evaluated using PROCHECK [28], "WHAT IF" [29], and VERIFY3D [27]. Results are shown in Table 2 A/B and additional file 5. The Ramachandran plot is correct, and according to theses programs, scores are within expected ranges for well-refined structures. Nevertheless, several residues located in flexible loops fall into disallowed regions of the Ramachandran plot (additional file 2A): Ser 100, Val 255, Thr 256 and Cys 215. The Ramachandran Plot statistics given by PROCHECK (additional file 2B) shows clearly that 99% of the residues are in allowed regions. The score corresponding to the chi-1/chi-2 angles of all residues is within expected ranges for well-refined structures (Table 2). The model has a normal distribution of residue types over the inside and the outside of the protein. Again, the backbone conformation analysis gives a score that is normal for correctly refined protein structures. The RMS Z-score given in Table 2 is expected to be around 1.0 for a normally restrained data set, and this is indeed observed as in the case of high-resolution X-ray structures. In the GBV-C polymerase model, bond angles and lengths can be considered to deviate normally from the mean standard bond angles.

**Table 2:** Quality of the model. A: Parameters reflecting the quality of the model checked by «WHAT IF» [29]. B: Quality of chain of the model. The model is verified at 2Å resolution. Parameter values in the table represent observed values for the GBV-C polymerase model compared with typical values obtained for well refined structures at the same resolution [28].

**A**					
Structure Z-scores:					

1st generation packing quality	-1.577				
2nd generation packing quality	-2.94				
Ramachandran plot appearance	-0.74				
chi-1/chi-2 rotamer normality	-0.224				
Backbone conformation	-0.904				
					
RMS Z-scores, should be close to 1.0:					

Bond lengths	0.950				
Bond angles	1.426				
Omega angle restraints	-0.923				
Inside/Outside distribution	1.096				
					
**B**					

Stereochemical parameter	N° of data	points Parameter	valueTypical	valueBand width	N° of bandwidth

Stereochemistry of main-chain					
Percentage residues in A, B, L	438	89	83.8	10	0.5
Omega angle S.D	507	6.8	6	3	0.3
Bad contacts 100 residues	3	0.6	4.2	10	-0.4
Zeta angle S.D.	476	2.8	3.1	1.6	-0.2
Hydrogen bond energy S.D.	305	0.7	0.8	0.2	-0.4
Stereochemistry of side-chain					
Chi-1 gauche minus S.D.	79	15.7	18.1	6.5	-0.4
Chi-1 trans S.D.	103	13.2	19	5.3	-1.1
Chi-1 gauche plus S.D.	205	11.4	17.5	4.9	-1.2
Chi-1 pooled S.D.	387	13	18.2	4.8	-1.1
Chi-2 trans S.D.	114	15.7	20.4	5	-0.9

As expected with such good scores, the model of the GBV-C polymerase is similar to that of HCV, and displays the essential features of the typical RNA dependent RNA polymerase fold (Figure 4A and 4B). However, we note two small differences between the HCV structure and the GBV-C model. First, Cys 283 and Cys 308 are spatially close enough to model a disulphide bridge (Figure 3 and additional file 3). This bond connects the fingers and the palm, and may stabilize the protein. Second, the superimposition of the GBV-C model and the HCV structure (Figure 4C) shows little but notable differences in the palm and thumb. The secondary structure elements are conserved in place and type, but they are shorter in the model than in the structure. These secondary structure elements should have similar functions, though. For example His 428 overlaps Tyr 448 of the HCV flap (Figure 4D) and replacement of the aromatic ring of the tyrosine by the histidine ring could play the same role during initiation (see discussion below).

### Surface analysis

**Figure 5:**
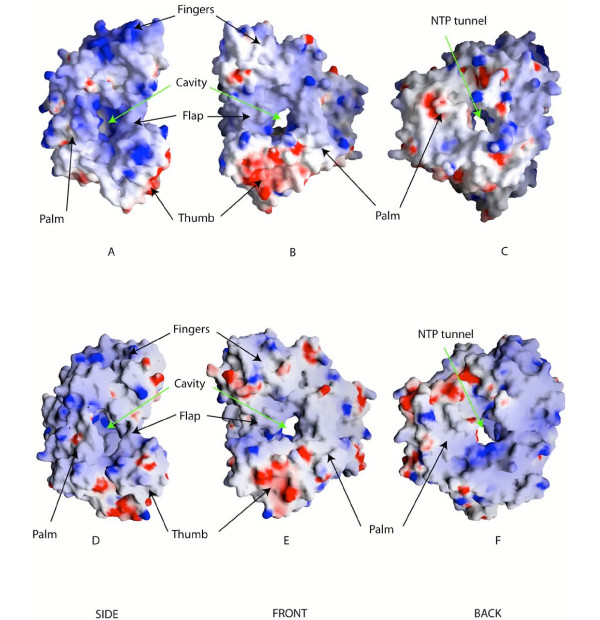
**Surface comparison of GBV-C and HCV RdRps**. **A, B, C **correspond to different views of the GBV-C polymerase surfaces calculated using GRASP. The surface is colored according to the electrostatic potential. The red correspond to negative charges, the white is neutral, and the blue corresponds positive charges. **D, E, F **correspond to the surface of HCV polymerase in similar and respective orientations. The color ramp is the same as for the GBV-C polymerase surface.

We note several differences between the surface shapes (Figure 5) of HCV RdRp and the GBV-C model. As the two backbones are superimposed these differences are only due to the variability of side chains. The sequence conservation reported for the GBV-C model (additional file 3) shows that amino acids oriented toward the inner side of the protein are conserved whereas the amino acid which are pointing to the surface show low identity. This surface variability may be explained by the fact that the GBV-C polymerase form a complex with other viral proteins, as it is the case for the HCV polymerase which interacts with NS3 or NS5A proteins, or as observed in the case of the poliovirus polymerase [30]. These other viral proteins may differ in their NS5B binding domain between HCV and GBV-C. Moreover, it has been shown that the HCV polymerase dimerizes and can form higher order structures after oligomerization. This multimerization is required for the HCV polymerase activity [31]. As the GBV-C polymerase is similar to HCV polymerase, the same oligomerization may also occur in the case of the GBV-C polymerase. Surface amino-acids have then to be specific to the virus to allow correct dimerization of the polymerase and/or interaction with the other components of the replicative complex. The electrostatic potential comparison is presented in Figure 5. It shows that the charges distribution on the surface of the model is globally equivalent to those located on the surface of HCV polymerase. We observe that the thumb in both cases is negatively charged (Figure 5B and 5E). The positive channel supposed to guide the RNA template to the catalytic site is very well conserved, and the flap is partially obstructing this cavity. The difference appears near the NTP tunnel (Figure 5C and 5F). In the HVC polymerase structure, the surface is clearly positively charged whereas in the GBV-C polymerase model the positive charge is less apparent.

### NTP-binding sites

In the HCV polymerase, the allosteric site forms a pocket where GTP binds. Such a pocket does exist in GBV-C despite sequence variability (Figure 3), and is located behind the thumb subdomain. The surface analysis shows that the pocket has a hydrophobic nature, except for the side chains of Asp 30 and Lys 473 that may however participate in the binding of a GTP molecule (see below).

In the HCV structure, several NTP molecules can bind to the catalytic site at P, C, and I sites. Indeed, up to 9 phosphate moieties can be seen in the crystal structure. Only the nucleotide bound at the C site is well defined, although its nucleobase is probably incorrectly located in the absence of the RNA template [8]. Clearly, a better definition of nucleotides and template is needed to understand the RNA synthesis process. On the other hand, the BVDV polymerase structure in complex with GTP in the catalytic cavity suggests a role for this nucleotide in the initiation of RNA synthesis, as proposed below.

### Docking of GTP in GBV-C

**Figure 6:**
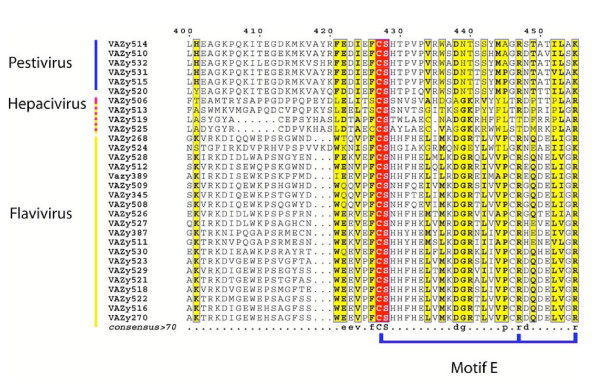
**General alignment of the E motif in *Flaviviridae *RdRps**. The conserved motif is labeled according to the nomenclature described for the RNA polymerase family. Invariant residues are highlighted in red, while conserved residues are boxed yellow highlighted in bold. Consensus sequence with 70% similarity is shown down the alignment. The sequences are sorted by genera.

**Figure 7:**
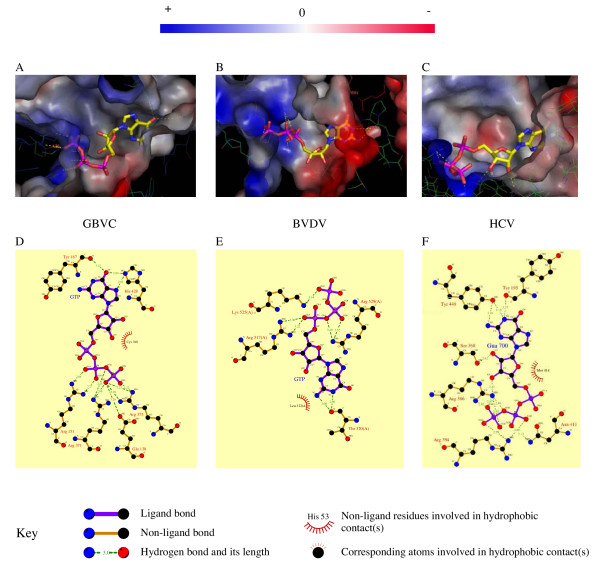
**Docking a GTP in the GBV-C polymerase**. A to C: Views of GTP-binding pockets. The surface is colored according to the electrostatic potential nomenclature. Hydrogen bonds are indicated in dotted lines and the numbering indicates the distance (in Å) between amino acids. A: The proposed GTP pocket in the GBV-C polymerase model with a docked GTP molecule. B: The BVDV polymerase structure in which a GTP molecule is co-crystallized. C: The proposed GTP pocket in the HCV polymerase structure with a docked GTP molecule. D to F: LIGPLOT presenting residues involved in the stabilization of GTP. Hydrogen bonds are indicated in dotted lines and the numbering indicates the distance (in Å) between amino acids. D: View of the GBV-C GTP pocket. E: same view of the BVDV GTP pocket. F: LIGPLOT of the HCV GTP pocket. Images were generated using PYMOL.

The analysis of the thumb in terms of structure and sequence comparison proved to be informative to propose an RNA synthesis initiation mechanism. Previously, in HCV polymerase the E motif has been proposed as a part of the site that accommodates the first NTP incorporated during initiation of RNA synthesis (P site). Motif E is defined by the CS-18X-R signature (Figure 3 and additional file 1) [8]. In the case of BVDV, the polymerase structure has also been solved in complex with GTP [12]. This GTP is found in a binding pocket that is mainly constituted by amino acids within motif E. Their side chains effectively stabilize the phosphate chains of GTP with an Arginine (Arg 529) further away in the sequence. The NS5B sequence comparison of *Flaviviridae *showed that motif E could be extended to CS-18X-[RKT]-x(8)-[RK] as a signature sequence (Figure 6). In the BVDV polymerase structure, the GTP molecule has been compared to a vestigial RNA molecule acting as a primer [12]. In the HCV polymerase structure, this GTP position corresponds to a cavity filled with water molecules. In the GBV-C model such a pocket exists, but its shape is different. Based on the GTP localization in the BVDV polymerase structure, we have docked a GTP molecule in GBV-C and HCV polymerase structures to see if these pockets could accommodate a GTP molecule in a similar manner. These three pockets are similar regarding position and nature of the conserved residues. This characteristic allows a perfect fitting of the molecule into the GBV-C and HCV pockets (Figure 7). In all cases, part of the cavity is positively charged contributing to the stabilization of the GTP-phosphate chain in the pocket. This stabilization involves Thr 367 and Arg 371 in GBV-C motif E and the corresponding Arg 386 and Arg 394 in HCV. The Ser 349 in GBV-C (Ser 367 for HCV) of the CS motif forms the bottom of the cavity. In the structure and both models, the cavity is obstructed by a proline (Pro 189 GBV-C; Pro 321 BVDV; Pro 197 HCV). However, amino acids stabilizing the guanine base are different. While the base is stabilized only by hydrogen bonds with Tyr 187 in GBV-C and Tyr 195 in HCV, Thr 320 and Tyr 581 stabilize it in the case of BVDV. In GBV-C and HCV an aromatic residue located at the extremity of the flap, His 448 in GBV-C and Tyr 448 in HCV forms the top of the cavity stabilizing the cycle of the base (Figure 7). Although the pocket is conserved in charged residues, the GTP position in the pocket is different. Indeed, because the GBV-C pocket is somehow smaller than in the case of BVDV, the GTP ribose is flipped and the phosphate chain bends to follow the surface of the pocket (Figure 7, compare A and B). In HCV polymerase, the cavity is larger than the GBV-C pocket and therefore the binding of the GTP molecule is closer to what is observed in the case of the BVDV polymerase structure (Figure 7 compare B and C).

**Figure 8:**
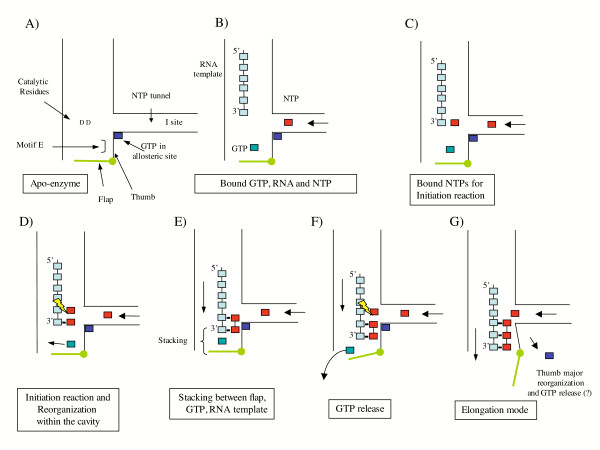
**A model for *de novo *RNA synthesis at the hepacivirus NS5B active site**. A: The polymerase is represented schematically to illustrate key points in the reaction mechanism. B: The RNA template is represented as clear blue squares. NTP are as red squares, the allosteric GTP is represented as a dark blue square, and the bound GTP as a green blue square. C: Binding of the first NTP in the active site. D: The initiation reaction is presented with a yellow lightening. Upon incorporation of the third NTP, the template and the neo synthesized RNA slide to the cavity pushing the GTP towards the flap. E: Intermediate position where the flap, GTP and RNA template are stacked. F: Opening of the flap and release of GTP. G: The polymerase shifts to the elongation mode; the thumb moves to fully open the cavity, and the elongation resumes.

Based on our docking results, we propose that motif E is the signature sequence of a GTP binding site in which GTP is required to hold the initiation complex tight. In our structural model, the GTP itself is too remote to act as a platform for the nucleotide positioned at the P site. The modeled GTP binding site together with the observed position of the flap lead us to suggest a mechanism for *de novo *initiation (Figure 8). We propose that once the first reaction of initiation is achieved (Figure 8C and 8D), the initiated template enters the pocket where the motif E GTP is located, and stacks against the guanine base (Figure 8E). This stacking induces a rearrangement of the base, which now contacts the flap. This latter interaction induces the opening of the flap leading to GTP release and further major structural changes within the polymerase (Figure 8F and 8G). The movement of the flap is supposed to occur to open the cavity allowing the elongation of the neo-synthesized RNA. The opening of the cavity implies that the thumb moves. It has been already observed that the fingers and the palm rotate as rigid body around the axis against the thumb domain [9]. In our model, the flap is spatially conserved suggesting that the same movement may occur during the elongation step of the GBV-C polymerization. Additionally, the position of the amino acid closing the cavity of the polymerase (flap in the case of GBV-C and HCV, or the β-sheet in the case of BVDV) suggests that the opening movement is specific for each virus. This movement would be best described as an opening from the top for HCV and GBV-C and, lateral for BVDV. Recently, we have characterized the initiation steps of RNA synthesis kinetically [32]. It is interesting to note that our present model is in agreement with the kinetic data showing that the N_2 _to N_3 _polymerization reaction is strongly rate limiting, and corresponds to the first partial opening of the flap to release GTP as proposed in Figure 8 panel F, whereas the other rate-limiting step from N_4 _to N_6 _corresponds to the other complete flap opening allowing dsRNA to exit from the active site as proposed in panel G.

## Conclusion

The recently published high-resolution three-dimensional structure of BVDV and HCV polymerase has allowed the structural comparison of the two polymerases. Major differences in fingers and thumb suggest that molecular interactions during the initiation mechanism are different. BVDV has been used as a model in the study of hepaciviruses. However, phylogenic analysis shows that GBV-C is more closely related to HCV than BVDV. We propose here a reliable model of the GBV-C polymerase structure.

The model of the GBV-C polymerase is poorly defined in loopy regions where most of the gaps have been introduced. Despite this imprecision, the very good scores of the structural indicators make us very confident of the reliability of our model. Moreover, the model is consistent with the known three-dimensional structure of RNA dependent RNA polymerases, and show conservation of all structural elements involved in polymerization (catalytic site, RNA positive channel, NTP tunnel). As expected after the alignment and prediction study, the GBV-C model is very close to the HCV structure, even with a conserved allosteric GTP binding site. Based on the BVDV polymerase/GTP complex structure, we generated a model of a corresponding complex of GBV-C. We propose a role for the GTP molecule bound at a site involved in the initiation of RNA synthesis. Our study provides useful information of the location of residues involved in the polymerization process and hence presents a useful resource for future biochemical analysis and drug discovery.

## Methods

### Sequence Retrieval

The sequences related to the different kind of polymerase were retrieved with a PSI-BLAST [18] with standard parameters from the public available protein database Swiss-Prot [33], Protein Data Bank (PDB) [34] and VaZyMolO [17]. For this study we have used different structures of HCV (PDB code: [1GX5, 1GX6]), and BVDV (PDB code: [1S48, 1S49]).

### Sequence alignment comparison

Alignment of representative sequences from several members of *Flaviviridae *were performed using CLUSTALW [35] with the following parameter. Slow Algorithm, Identity matrix for pairwise alignment and BLOSUM series matrix for multiple alignments. The alignment was then carefully analyzed and optimized with SEAVIEW [36], taking into account the secondary structure prediction and structural elements when existing. This alignment was cross checked using 3DJURY [37].

The secondary structure predictions were carried out using JPRED^2 ^[38], PSI-PRED [20] and PREDICT-PROTEIN Server [19]. We used PREDICT-PROTEIN with a window of 150 amino acids in order to increase the sensitivity of the prediction. 20 amino acids overlap with each common superimposed window. The results presented are consensus. Sequence alignment with structural information (structure or predictions) and the comparison of the structure one dimension of the known viral polymerases was performed using ESPript 2.0 [39] and ENDscript 1.0 [40].

To visualize conserved region in amino acids composition on the reference structure, we used BOBSCRIPT [41]. The similarity scores were calculated from the CLUSTALW [35] alignment and they are shown on this structure with a white (low score) to red (identity) color ramp.

### Phylogenetic analysis

The sampling variance of the distance values was estimated from 1000 bootstrap resamplings of the alignment columns. The evolutionary inference was performed according to the Neighbor-joining method. Multiple runs were conducted with randomized sequence input order to avoid the tree being caught in a local statistical minimum. The tree was generated using Phylodendron (^©^1997 Gilbert).

### Model building, refinement and evaluation

The resulting multiple sequence alignment with the consensus secondary structure prediction was used as template to generate the threading alignment. The derived pairwise alignment serves as reference for preparing the file for the model. SWISS-PDB VIEWER [21] was used to generate a first threading model. The three dimensional model of the GBV-C RdRp was constructed using the crystal structure coordinates of the HCV polymerase [8,7] (PDB code: 1GX5, 1QUV). Main gaps appear in loops and smaller ones in helices. This alignment and the threading model serve as a template file for SWISS-MODEL [21]. The non-modeled loops were manually built after scanning the loop database. The model was then minimized with a cut off of 10 Å with 40 cycles of steepest descent until the gradient fell below 10 Kcal/mol and 20 cycles of conjugate gradient. The computations were done in vacuum with using GROMOS 96 [42,43] force field. To generate alternate models, we have used the 3D-JIGSAW [23-25] server, SCRWL [22] and MODELLER [26]. In this latter, positions of predicted catalytic residues and secondary structure elements were used as spatial restraints.

Surface comparison of the template and the model were performed with GRASP [44]. The generated models were checked using PROCHECK [285] "WHAT IF" [29] and/or VERIFY3D [27].

### Docking GTP molecule in GBV-C

The 3D model of the GBV-C RNA polymerase was used as a target for the docking of GTP. We first superimposed the structure of BVDV RNA polymerase/GTP complex (PDB code 1S49) with our 3D model. This step was performed with the program Turbo-Frodo [44]. A docking study was performed to explore the presence or absence of a GTP binding pocket like, as it was described in the BVDV polymerase structure. For the docking procedure, the program AUTODOCK 3.0.5 [45] was used with a grid spacing of 0.375 Å and 40 × 40 × 40 number of points. The grid was centered on the mass center of the GTP molecule. The GA-LS method was adopted using the default settings. Amber united atoms were assigned to the protein using the program AUTODOCK TOOLS. 250 possible binding conformations were generated. The results of AUTODOCK run were clustered using a RMSD tolerance of 1.0 Å. We considered the structure of the first cluster. To validate the use of the AUTODOCK program, the docking study was performed on the BVDV polymerase with GTP as a reference. This program successfully reproduced the experimental binding conformation with acceptable root-mean-square deviation (RMSD) of atom coordinates. Finally, the interaction models of GTP with the binding pocket were produced using the LIGPLOT program [46].

## Authors' contributions

FF carried out the sequence retrieval, alignments, modeling, phylogenic studies, and, the structure and docking analysis. CB and HD performed the docking and structure analysis. BC conceived of the study, and participated in its design, analysis, and coordination. FF, CB, HD and BC all contributed to writing the final manuscript and interpretation of data.

## Note

Table 1 – A listing of Flaviviridae

Viruses used in the study, together with their correspondent VaZyMolO and NCBI accession numbers.

Table 2 – Quality of the model

A: Parameters reflecting the quality of the model checked by « WHAT IF » [26].

B: Quality of chain of the model. The model is verified at 2Å resolution. Parameter values in the table represent observed values for the GBV-C polymerase model compared with typical values obtained for well refined structures at the same resolution [25].

## Supplementary Material

Additional File 1 **Multiple alignment of *Flaviviridae *RNA polymerase palm subdomains**. The conserved motifs are labeled according to the nomenclature described for the RNA polymerase family. Invariant residues are highlighted in red, while conserved residues are boxed yellow highlighted in bold. A consensus sequence with 70% similarity is shown below the alignment. The sequences are sorted by genera. Click here for file

Additional File 2**Ramachandran plot of the GBV-C Model with PROCHECK statistics**. A: Ramachandran plot of GBV-C polymerase model. Favoured and allowed regions are in red and yellow, respectively. All residues are represented by black boxes (■) except glycine (▲). Red boxes () highlight residues in forbidden regions.Click here for file

Additional File 4 **Superimposition of the models generated with different programs**. A. Models generated using SWISS-MODEL (represented in light blue), and MODELLER: model 1 (represented in light green), model 3 (represented in magenta) or model 2 (represented in yellow) were superimposed. B. 90° rotation view of the same superimposed models. C. Zoom view of the superimposed amino acids of the GTP pocket. Click here for file

Additional File 5 score of the different model generated according to VERIFY3D. Click here for file

Additional File 3 **Residues conservation plotted on the structure**. Calculated homology based on the superimposition of the structure of HCV polymerase on the GBV-C polymerase model. The figure was done using BOBSCRIPT. The similarity is shown on this structure by a white (low score) to red (identity) colour ramp. The green doted line indicates the position of the disulfide bridge. Click here for file
