# Supplementary material for: The modeled structure of the RNA dependent RNA polymerase of GBV-C Virus suggests a role for motif E in Flaviviridae RNA polymerases
Source: BMC Bioinformatics. 2005 Oct 14;6:255. doi: 10.1186/1471-2105-6-255 (PMC1283970; doi:10.1186/1471-2105-6-255)
Supplement: Additional File 1 — Multiple alignment of Flaviviridae RNA polymerase palm subdomains. The conserved motifs are labeled according to the nomenclature described for the RNA polymerase family. Invariant residues are highlighted in red, while conserved residues are boxed yellow highlighted in bold. A consensus sequence with 70% similarity is shown below the alignment. The sequences are sorted by genera. [file 1471-2105-6-255-S1.pdf]

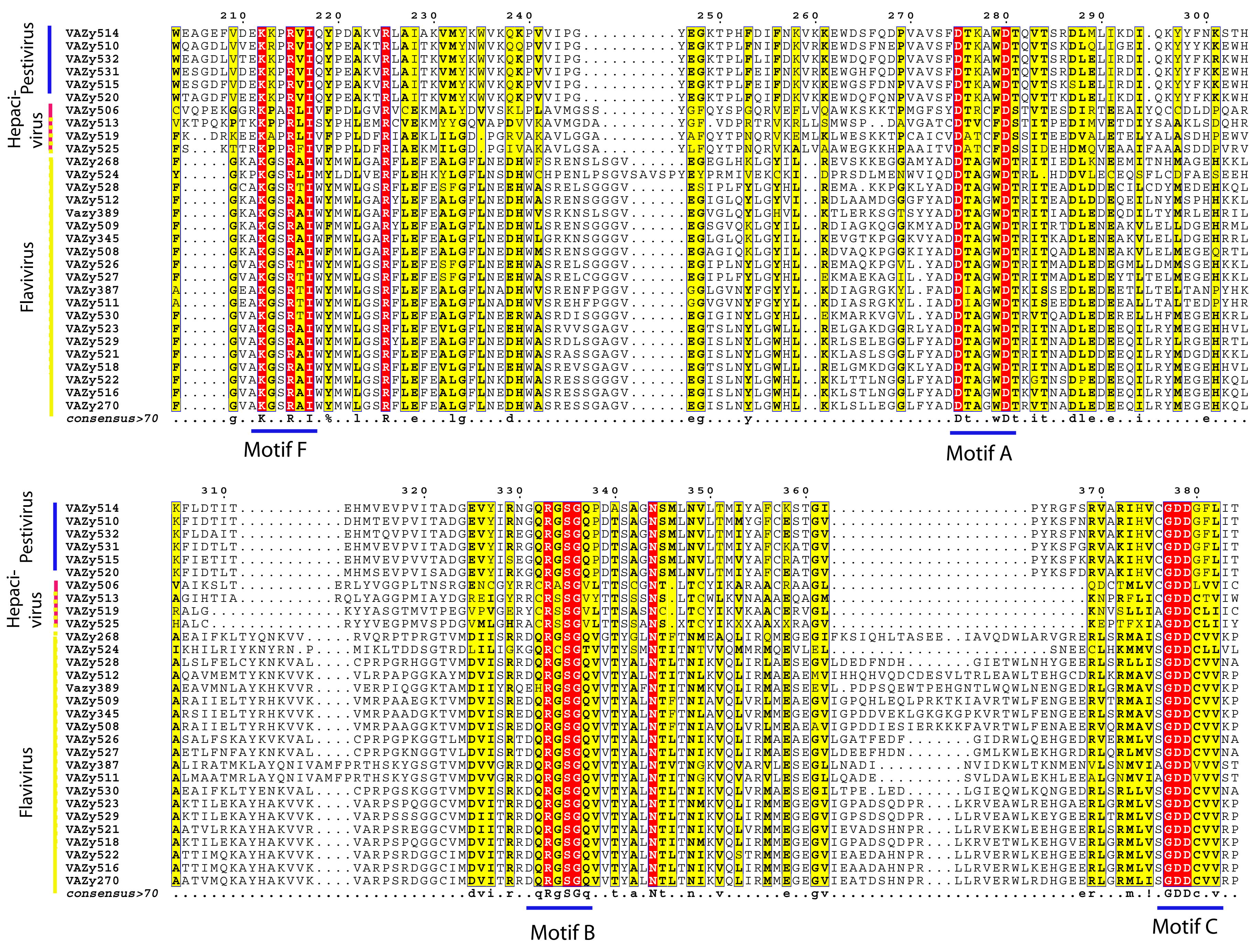

Pestivirus
Hepaci-virus
Flavivirus
210 220 230 240 250 260 270 280 290 300
VAZy514
VAZy510
VAZy532
VAZy531
VAZy515
VAZy520
VAZy506
VAZy513
VAZy519
VAZy525
VAZy268
VAZy524
VAZy528
VAZy512
Vazy389
VAZy509
VAZy345
VAZy508
VAZy526
VAZy527
VAZy387
VAZy511
VAZy530
VAZy523
VAZy529
VAZy521
VAZy518
VAZy522
VAZy516
VAZy270
consensus>70
Motif F
Motif A
310 320 330 340 350 360 370 380
consensus>70
Motif B
Motif C
