# Supplementary material for: The modeled structure of the RNA dependent RNA polymerase of GBV-C Virus suggests a role for motif E in Flaviviridae RNA polymerases
Source: BMC Bioinformatics. 2005 Oct 14;6:255. doi: 10.1186/1471-2105-6-255 (PMC1283970; doi:10.1186/1471-2105-6-255)
Supplement: Additional File 2 — Ramachandran plot of the GBV-C Model with PROCHECK statistics. A: Ramachandran plot of GBV-C polymerase model. Favoured and allowed regions are in red and yellow, respectively. All residues are represented by black boxes (■) except glycine (▲). Red boxes () highlight residues in forbidden regions. [file 1471-2105-6-255-S2.pdf]

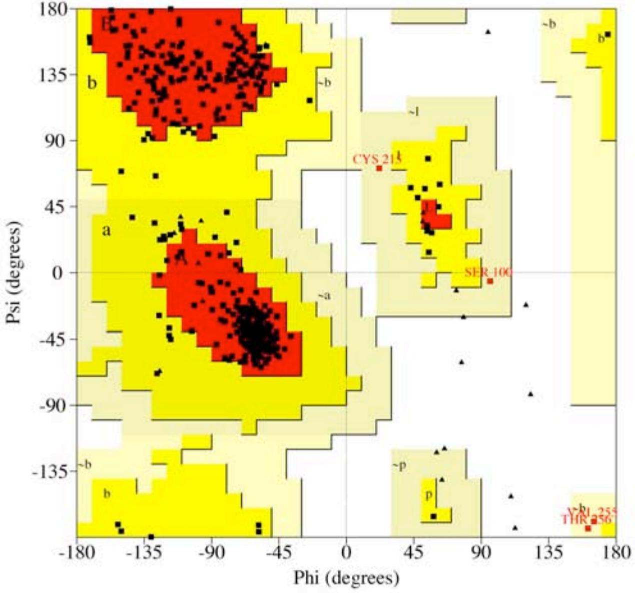

A

Plot statistics

Residues in most favoured regions [A,B,L]	390	89.0%
Residues in additional allowed regions [a,b,l,p]	44	10.0%
Residues in generously allowed regions [~a,~b,~l,~p]	4	0.9%
Residues in disallowed regions	0	0.0%
	----	------
Number of non-glycine and non-proline residues	438	100.0%
Number of end-residues (excl. Gly and Pro)	2	
Number of glycine residues (shown as triangles)	34	
Number of proline residues	36	

Total number of residues	510	

B
