# Supplementary figures and images for: The modeled structure of the RNA dependent RNA polymerase of GBV-C Virus suggests a role for motif E in Flaviviridae RNA polymerases
Source: BMC Bioinformatics. 2005 Oct 14;6:255. doi: 10.1186/1471-2105-6-255 (PMC1283970; doi:10.1186/1471-2105-6-255)

A

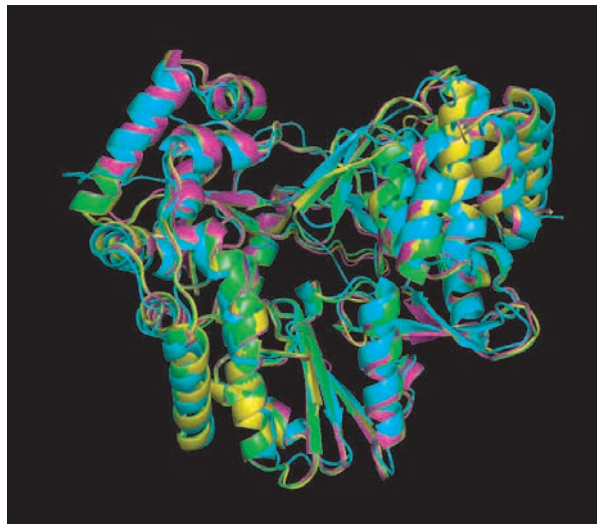

B

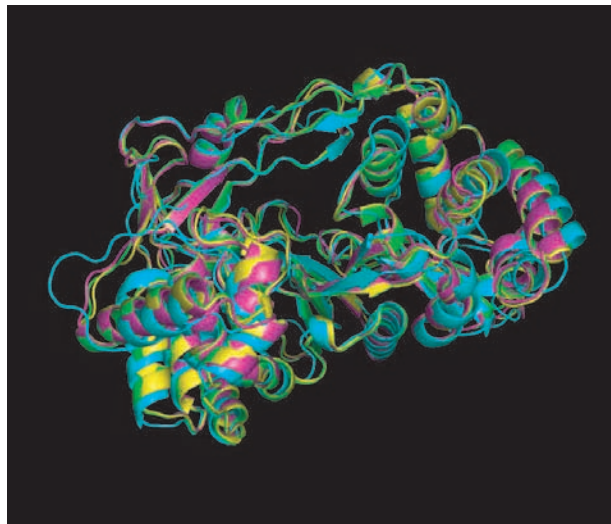

C

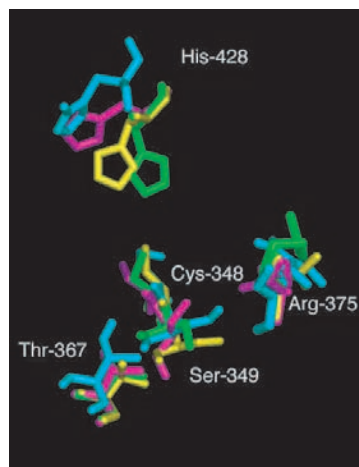

Supplement: Additional File 4 — Superimposition of the models generated with different programs. A. Models generated using SWISS-MODEL (represented in light blue), and MODELLER: model 1 (represented in light green), model 3 (represented in magenta) or model 2 (represented in yellow) were superimposed. B. 90° rotation view of the same superimposed models. C. Zoom view of the superimposed amino acids of the GTP pocket. [file 1471-2105-6-255-S4.pdf]

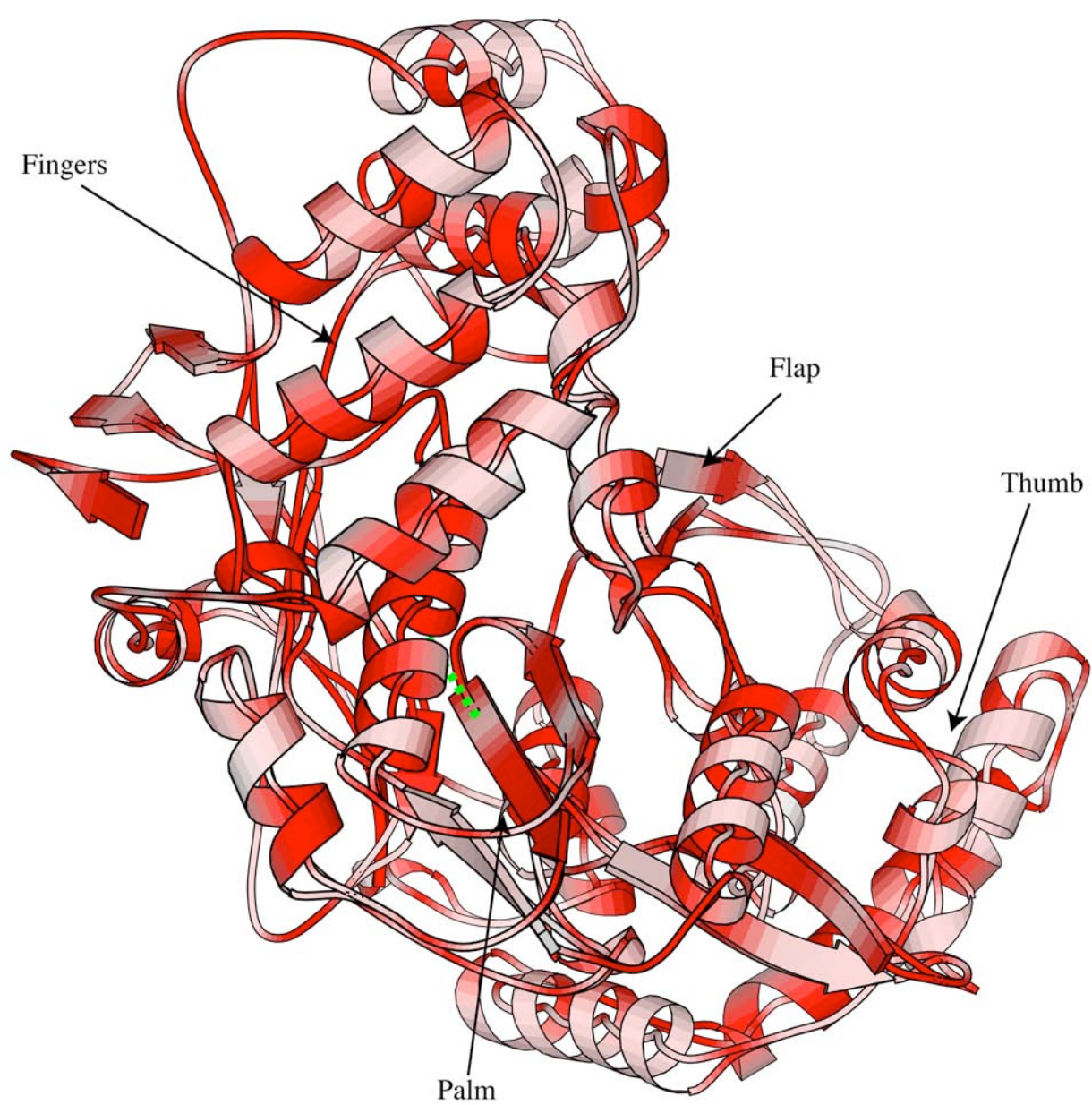

Supplement: Additional File 3 — Residues conservation plotted on the structure. Calculated homology based on the superimposition of the structure of HCV polymerase on the GBV-C polymerase model. The figure was done using BOBSCRIPT. The similarity is shown on this structure by a white (low score) to red (identity) colour ramp. The green doted line indicates the position of the disulfide bridge. [file 1471-2105-6-255-S3.pdf]
