# Supplementary material for: The modeled structure of the RNA dependent RNA polymerase of GBV-C Virus suggests a role for motif E in Flaviviridae RNA polymerases
Source: BMC Bioinformatics. 2005 Oct 14;6:255. doi: 10.1186/1471-2105-6-255 (PMC1283970; doi:10.1186/1471-2105-6-255)
Supplement: Additional File 5 — score of the different model generated according to VERIFY3D. [file 1471-2105-6-255-S5.pdf]

Program	model	Verify3D	Remark
Swissmodel		0,345	510aa problem localized between residues 100-118
Modeller	1	0,38	
	2	0,36	
	3	0,35	
3D-JiGSAW		0,35	ONLY the core 414aa
		0,37	ONLY the core 405aa
		0,32	ONLY the core 420aa
